# Assessing the efficacy of molecularly targeted agents on cell line-based platforms by using system identification

**DOI:** 10.1186/1471-2164-13-S6-S11

**Published:** 2012-10-26

**Authors:** Xiangfang Li, Lijun Qian, Jianping Hua, Michael L Bittner, Edward R Dougherty

**Affiliations:** 1 Department of Electrical and Computer Engineering, Texas A&M University, College Station, TX 77843, USA; 2 Department of Electrical and Computer Engineering, Prairie View A&M University, Prairie View, TX 77446, USA; 3 Computational Biology Division, Translational Genomics Research Institution, Phoenix, AZ 85004, USA; 4 Department of Bioinformatics and Computational Biology, University of Texas M.D. Anderson Cancer Center, Houston, TX 77030, USA

## Abstract

**Background:**

Molecularly targeted agents (MTAs) are increasingly used for cancer treatment, the goal being to improve the efficacy and selectivity of cancer treatment by developing agents that block the growth of cancer cells by interfering with specific targeted molecules needed for carcinogenesis and tumor growth. This approach differs from traditional cytotoxic anticancer drugs. The lack of specificity of cytotoxic drugs allows a relatively straightforward approach in preclinical and clinical studies, where the optimal dose has usually been defined as the "maximum tolerated dose" (MTD). This toxicity-based dosing approach is founded on the assumption that the therapeutic anticancer effect and toxic effects of the drug increase in parallel as the dose is escalated. On the contrary, most MTAs are expected to be more selective and less toxic than cytotoxic drugs. Consequently, the maximum therapeutic effect may be achieved at a "biologically effective dose" (BED) well below the MTD. Hence, dosing study for MTAs should be different from cytotoxic drugs. Enhanced efforts to molecularly characterize the drug efficacy for MTAs in preclinical models will be valuable for successfully designing dosing regimens for clinical trials.

**Results:**

A novel preclinical model combining experimental methods and theoretical analysis is proposed to investigate the mechanism of action and identify pharmacodynamic characteristics of the drug. Instead of fixed time point analysis of the drug exposure to drug effect, the time course of drug effect for different doses is quantitatively studied on cell line-based platforms using system identification, where tumor cells' responses to drugs through the use of fluorescent reporters are sampled over a time course. Results show that drug effect is time-varying and higher dosages induce faster and stronger responses as expected. However, the drug efficacy change along different dosages is not linear; on the contrary, there exist certain thresholds. This kind of preclinical study can provide valuable suggestions about dosing regimens for the *in vivo *experimental stage to increase productivity.

## Introduction

Drug development is currently an expensive and prolonged process with high attrition rate. The rate of new drug approvals in the U. S. has remained essentially constant since 1950, while the costs of drug development have soared [1]. Industry analysts estimate that it takes $1 billion to $4 billion in R&D and 10-15 years for every new drug brought to market [1-3]. In aggregate, the industrial average rate of attrition measured from first trials in humans to registration seems to be locked at ~85-90% [4,5]. The situation in oncology drug development is even worse [3,6,7]. By contrast, the overall clinical success rate for new anticancer agents (~5%) is much lower than other therapeutic areas (e.g. success rate for cardiovascular diseases is ~20%) [8]. As a result, the American Cancer Society's 2005 statistical report shows that cancer is now the leading cause of death for Americans under age 85 [9]. One common explanation for the recent shrinking of oncology drug pipelines is that discovery is moving into more complex areas of human health [10,11], such as cancer, which is more likely to result from the interaction of several different genes/pathways [12,13]. The conundrum confronting the cancer research community is twofold: first, the pharmaceutical industry is facing difficult times owing to low productivity and spiraling cost [4]; second, on consumers front, patients await better treatments and cancer drugs are an unaffordable luxury for many consumers [14]. To move ahead, scientists realize that they need some fresh thinking in basic, translational and clinical research [15] to improve R&D productivity and reduce attrition rates, and such efforts calls for joint collaboration from different disciplines [5,16-20].

The focus of anticancer drug development in recent years has shifted from cytotoxic drugs to targeted therapy [16,19,21-23]. The goal of this target-based approach is to improve the efficacy and selectivity of cancer treatment by developing agents that block the growth of cancer cells by interfering with specific targeted molecules needed for carcinogenesis and tumor growth [21,22]. This approach is different from traditional cytotoxic anticancer drugs, where most compounds are targeted against molecules required for the maintenance of structural and genetic integrity of rapidly dividing cells. However, despite advances in understanding of the molecular mechanisms of cancer, the promise of targeted cancer therapy remains largely unfulfilled [8,24], with only a few well-known examples, such as imatinib [25] and trastuzumab [26], currently approved [27]. Many promising candidates prove ineffective or toxic owing to a poor understanding of the molecular mechanisms of biological systems they target. Different reasons have been proposed to explain this limited effectiveness of anticancer drug development, including insufficient translational research and lack of adequate preclinical models that recapitulate disease complexity and molecular heterogeneity [8,16,28,29]. Ideally, preclinical models should validate the target, provide information about the mechanism of action of the drug, and identify pharmacodynamic markers of activity. Once the target and mechanism of action have been identified using *in vitro *models, experiments should be undertaken to ensure that inhibition of the target can be achieved at tolerated doses *in vivo *and to identify possible biomarkers of response. Improved preclinical evaluation of compounds has the potential to augment the detection of activity and toxicity, and to reduce the high attrition rate.

While the lack of specificity of the traditional cytotoxic anticancer agents allows a relatively straightforward, well-established approach, developing a paradigm to better analyze the efficacy of molecularly targeted agents (MTAs) is substantially more complex [18,22,30-32]. Many targets are involved in cell signaling pathways, which are most often not linear, but connected and redundant [33]. Control strategies typically involve a higher multiplicity of inputs and a multiple layer of feedback [34]. As a result, strategies traditionally applied to the development of cytotoxic drugs may not be appropriate for MTAs [32]. Current treatment plan and efficacy evaluations are usually designed empirically for MTAs, without adequate knowledge of the optimal dose and the appropriate schedule [32]. A novel preclinical model combining experimental methods and theoretical analysis is proposed in this study to investigate the mechanism of action and identify pharmacodynamic characteristics of the drug. It is expected that through such preclinical study, valuable suggestions about dosing regimens could be furnished for the *in vivo *experimental stage to increase productivity. We consider several challenges for MTA dosing.

**Figure 1:**
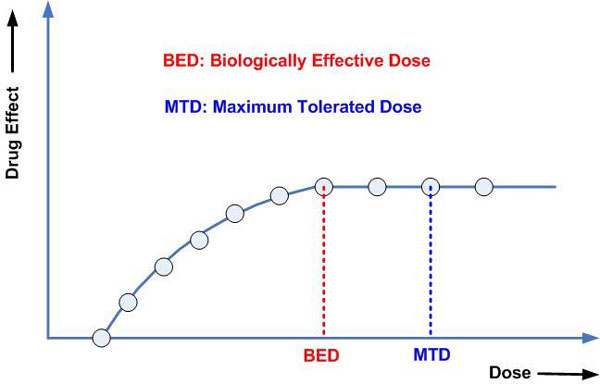
**A hypothetical dose-effect curve for targeted therapy**.

Firstly, the optimal dose has usually been defined as the "maximum tolerated dose" (MTD) for conventional cytotoxic anticancer drugs rather than the dose that produces a quantifiable therapeutic effect. This toxicity-based dosing approach is founded on the assumption that the therapeutic anticancer effect and toxic effects of the drug increase in parallel as the dose is escalated [22]. Such an assumption is sound if the mechanisms of action of the toxic and therapeutic effects are the same, as is often the case with cytotoxic agents. However, most MTAs are expected to be more selective and less toxic than conventional cytotoxic drugs [23]. As a result, the maximum therapeutic effect may be achieved at a dose, defined as the "biologically effective dose" (BED), which could be substantially lower than the traditionally established MTD as discussed by Johnston [31]. A hypothetical dose-effect curve is shown in Figure 1. In addition, the toxic effect may not parallel the therapeutic effect and not be predictive of the therapeutic effect [22]. Hence, the dosing study for MTAs should be based on both drug efficacy and toxicity considerations. Enhanced efforts to molecularly characterize the drug efficacy for MTAs in preclinical models will be valuable for successfully estimating the BED for clinical trials.

Secondly, the pharmacodynamics (PD) of drugs have been extensively investigated *in vitro *and *in vivo*; however, most analyses have reported the relationship of drug exposure to drug effect at a fixed time point. When drug effect is examined at a fixed time point, the drug concentration-effect relationship can be characterized through well established models, such as the Hill equation [35], also called the sigmoidal *E_max _*model [36]. However, characterization of the entire time course of drug effect may provide additional information [37]. For example, it may help to design the optimal schedule for drug administration.

Thirdly, traditional design of the dosing regimen to achieve some desired target goal such as relatively constant serum concentration may not be optimal because MTA targets mostly sit in interacting complex dynamical regulatory networks and such complex target contexts pose significant challenges for assessing mechanisms of action for MTAs [30]. For example, Shah and co-workers [38] demonstrate that the BCR-ABL inhibitor dasatinib, which has greater potency and a short half-life, can achieve deep clinical remission in CML patients by achieving transient potent BCR-ABL inhibition, while traditional approved tyrosine kinase inhibitors usually have prolonged half lives resulting in continuous target inhibition. A similar study of whether short pulses of higher dose or persistent dosing with lower doses have the most favorable outcomes has been carried out by Amin and co-workers in the setup of inactivation of HER2-HER3 signaling [39].

In sum, it is difficult and expensive to optimize dosing regimens using strictly empirical methods for MTAs. A novel preclinical model combining experimental methods and theoretical analysis is proposed in this study to investigate the mechanisms of action and identify pharmacodynamic characteristic of MTAs. As a first step, the time courses of drug effect for different doses are quantitatively studied on cell line-based platforms using system identification, where a tumor cell's response to investigational drugs through the use of fluorescent reporters is sampled frequently over a time course. A dynamic model is proposed to study the time course of drug efficacy for MTAs and then the experimental data are analyzed by our proposed model using a Kalman filter. Through such preclinical study, valuable suggestions about dosing regimens may be furnished for the *in vivo *experimental stage to increase productivity.

## Methods

The proposed approach is an integration of experiment and theory to investigate regulatory process dynamics by combining multiple complementary disciplines, including: (i) using fluorescent reporters in molecular technology to study cells' transcriptional activities under drug perturbation; (ii) these being captured by an automatic epifluorescent microscope over a time course; and (iii) such data being processed by large-scale image processing for dynamic analysis. A truly multi-dimensional dynamics of tumor cell response to drugs can be characterized through systematic perturbations to test different combinations of cell types, reporters, and drugs/dosages, augmented by iterative systematic theoretical analysis. This methodology differs from high-throughput technique like RNA expression profiling with microarrays, which provide a snapshot of an aspect of the system at one time point.

### Experimental methodology

Understanding cell response to a drug requires experimental designs that ask very specific questions about what is happening in a cell in the absence of a drug and how the cell activities change when the drug is present. The objective of the experimental protocol is to efficiently capture cell process dynamics in response to drugs and thereby obtain a deeper understanding of the genetic regulatory mechanisms, the point being to make preclinical research more predictive. Fluorescent reporters have long been used in molecular technology to study cells' transcriptional activities or the cellular localization of components, either in a population of cells or a single cell [40-42]. In this study, we track the transcriptional activities of particular genes. A fluorescent reporter to serve this purpose can be constructed by fusing the promoter region of a gene of interest with the coding sequence of a fluorescent protein, most commonly a green fluorescent protein (GFP). By delivering a single cassette bearing the promoter/GFP reporter into the genome of each cell in a population of cells, any change in the expression levels of the native coding sequence driven by that promoter will be reflected in the transcriptional activity of the cassette. This allows the estimation of the total fluorescence of the reporter in the cell, captured by imaging with an epifluorescent microscope, which is then used as a relative measure of the transcriptional activity of the native gene. Because this procedure is non-invasive to the cell, it allows tracking of the same cell population for an extended period of time by imaging the same site repeatedly. The recent introduction of automated digital microscopes allows researchers to use multi-well microtiter plates and sequentially capture the transcriptional activities in all wells. In our experimental protocol, a single assay is carried out by epifluorescent imaging of a site at the bottom of each well in a 384 well plate, producing an image of the cells in that region (~200-400 cells) bearing fluorescent reporters. The imaging speed of automated systems easily accommodates sampling an entire 384 well plate at hourly intervals. If needed, the experiment can be extended to multiple plates to cover a wider range of cell types and reporters.

In this experimental set-up, using different wells to test different combinations of cell type, GFP reporter and experimental condition allows this approach to provide a multi-dimensional examination of the cells' responses to a variety of stimuli. Not only can it follow multiple genes simultaneously, but it can also compare cellular activities under various conditions. Furthermore, it captures the dynamics of transcriptional regulation. This produces data on ~200-400 individual cells per well that can be analyzed both individually, as a distribution, or in aggregate, as an average. Fluorescent intensity data can be extracted from these images using specialized image analysis tools developed for this application [43]. This image processing procedures include finding cells, identifying individual cells, and quantifying the fluorescence associated with each cell. The objective is to extract gene expression levels from the fluorescent image and track them over the time course. We approach this goal through morphology-based image processing methods.

#### Image processing

**Figure 2:**
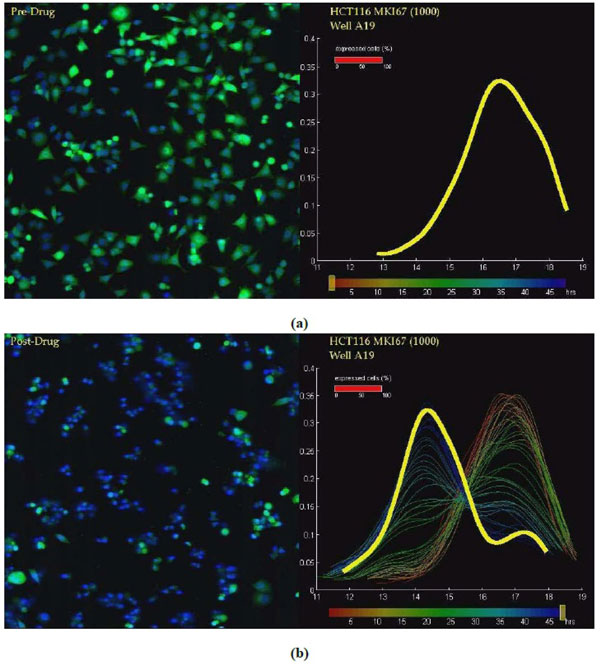
**Time course response to lapatinib by HCT116 with reporter for MKI67**: Left panels show 2 typical fluorescent images (nuclei: blue, GFP: green) sampled for the same site in a 48-hour lapatinib treatment. a) The upper panels show the case before any drug is applied. b) The lower panels show the case 48 hours after lapatinib was added. The right panels show the log2(GFP) intensity histogram for each time point. The fraction of the total population having a particular intensity is shown on the y-axis and the log2 intensity of the eGFP fluorescence measured for the cell is shown on the x-axis. The distribution before drug is shown as a thick yellow line at the upper right panel. The lower right panel shows the profile that is color-coded with time, starting with red, changing to yellow and then green, and ending with blue. The profile at the ending time points is shown with bold yellow line.

Typical fluorescent images are shown in Figure 2 (left panels), where nuclei are detected in the blue channel and promoter reporters to study cells' transcriptional activities are detected in the green channel. With a 384-well plate there will be at least 384 videos for evaluation and the number can be much higher if the experiment requires multiple plates to cover all experimental conditions. Visual evaluation is unreliable when one needs to quantitatively compare different conditions and the high-throughput nature of the green fluorescent protein reporter approach calls for a more automatic and quantitative solution to efficiently extract gene-expression levels from the fluorescent images and track them over the time course.

**Figure 3:**
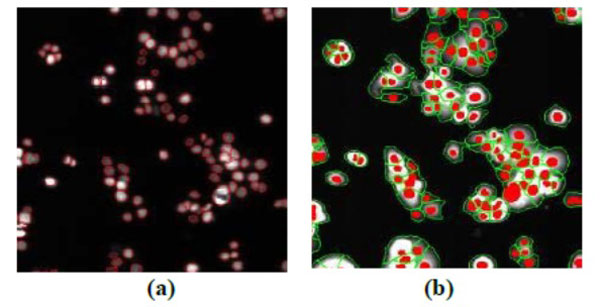
**Segmentation Results**: a) left panel: nuclear channel, where red lines are the identified nuclei boundaries; b) right panel: reporter channel, where green lines are identified cell boundaries, while the red objects are the nuclei used as markers.

To facilitate automatic processing of the experiment results, the transcriptional levels of the fluorescent images need be properly extracted, quantized, and saved and the image processing algorithm should be fast with good balance between performance and robustness [43]. An algorithm based on morphological image processing [44], in particular, the watershed transformation [45] is currently adopted in our study. Overall, the image processing breaks down into three major components: (i) nuclei channel segmentation, (ii) reporter channel segmentation, and (iii) measurement of cell-by-cell promoter activity levels. Figure 3 shows the segmentation results of a typical fluorescent image pair, where only a portion of the full image is shown in order to show the segmentation details. Once the individual cells are identified, the transcriptional activity represented by the reporter is extracted for every cell by summing up the background subtracted pixel intensity of the whole cell area and taking a log_2 _transform before being exported.

#### Experimental set-up for the dosing study

The dosing study is carried out on the colon cancer cell-line HCT116 with a reporter for the MKI67 gene, a nuclear antigen tightly correlated with proliferation [46,47], with responses to lapatinib treatment with 6 dosages (1 to 32 *µ*M). First we infect the HCT116 cell lines with the desired packaged reporter (packaged as lentiviral particles). Then plate cells/reporter pair in a media containing a live-cell nuclear stain. The cells are allowed to attach to the plate and grow overnight. Drugs are added to the appropriate wells (we have 6 wells [biological duplicates] for each dosage). In order to remove environmental effects, such as growth factor depletion, there are 6 control wells for each dosage (no drug added, total 36 wells). We image the plate once an hour for 48 hours to characterize the response of each cell/reporter pair to the drug over time. Note that the fluorescence intensity of cells without a GFP reporter expressed is not zero, since cells have numerous small molecules which fluoresce in the same wavelengths as GFP when excited with 488 nm light. This defines the minimum fluorescence, which is approximately 2^14^. One of the time courses from experiment (dosage = 8*µ*M) is shown in Figure 2. The left panels of Figure 2 show two fluorescent images sampled for the same site in a 48 hour lapatinib treatment for 8 *µ*M dosage. The right panels of Figure 2 show the log_2_(GFP) intensity histogram for each time point.

Since MKI67 is turned on during proliferation and off when the cells are not cycling, it is expected to show a binary, switch-like histogram of cell intensities, rather than a graded transition. This behavior is observed in Figure 2. We have the readout of the GFP intensity level for each individual cell/dosage pair with 48 time points. These can be compared with a threshold value to determine whether that cell is shifted or not [37,43]. Such a reporter assay allows one to determine the dynamics of drug responses for different dosages. Consequently, we propose a time-varying model for the cell shifting process where the drug effect coefficient is assumed changing with time. This is in contrast to many existing approaches where the drug effect coefficient is treated as a constant and the experiment just provides one reading rather than time-series characterization.

### Mathematical model formulation

The experimental results provide information on the percentage of cells shifted as a consequence of the drug activity. The measurements facilitate asking important questions in drug development. For instance, does dosing alter the extent of response, the timing of response, or both? In addition to qualitative questions, we are interested in modeling the drug effect *quantitatively*, which requires a novel mathematical model that is biologically sound and fits the experimental setup. Our experiments and the proposed modeling has two important features: (i) Our experiment is based on the readout of the intensity level of each individual cell, which is compared with a threshold value to determine whether that cell is shifted or not. Although we count the number of shifted cells at each sampling time point, the proposed model is *not *a population model merely giving the average readout of all the cells. (ii) Our experiment collects time-series data under drug perturbation for 48 hours, with one sample per hour. A time-varying model is proposed for the cell shifting process, where the drug effect coefficient is assumed changing with time.

Because there are different numbers of cells in different wells (the range is about ~200-400 cells per well), we perform normalization to calculate the percentage of cells shifted. Since there are many factors including drug effect that contribute to the cell shifting, calibration is performed by comparing to the control group to exclude other contributing factors. The notations used in this work are listed below

• *N*: total number of cells

• *N*_1_(*t*): number of shifted cells at time *t *after applying drug

• *ρ*_1_(*t*) = *N*_1_(*t*)/*N*: percentage of cells shifted at time *t *after applying drug

• *N_c_*: total number of cells in the control group (no drug applied)

• *N*_1*c*_(*t*): number of shifted cells at time *t *in the control group

• *ρ*_1*c*_(*t*) = *N*_1*c*_(*t*)/*N_c_*: percentage of cells shifted at time *t *in the control group

• *ρ*(*t*) = *ρ*_1_(*t*) − *ρ*_1*c*_(*t*): calibrated percentage of cells shifted at time *t *after applying drug

• *ρ_av_*(*t*) = *E*[*ρ*(*t*)]: mean of the calibrated percentage of cells shifted at time *t *after applying drug

• *X_i_*(*t*): state of cell *i *at time *t *after applying drug (either shift-ready or not)

We justify *N*_1_(*t*) being modeled as a Gaussian process when the number of cells per well is sufficiently large. Then a model is proposed for the cell shifting process, where the calibrated percentage of shifted cells follows a Gaussian process.

#### N_1_(t) is a Gaussian process when the number of cells per well is large enough

In general, *N *is a random variable since *N *may be different from well to well in the experiment; however, *N *can be treated as a known constant for each specific well, as can *N_c_*. At any given time point *t_j _*in the experiment, *X_i_*(*t_j_*) can be considered as either shift-ready or not. Thus, the experiment of drug effect on each cell can be treated as a Bernoulli trial and *X_i_*(*t_j_*) can be modeled as a Bernoulli random variable, i.e., the Probability Mass Function (PMF) of *X_i_*(*t_j_*) is given by

(1) $$P_{X_{i}}\left(x_{i}\right)=\left\{\begin{array}{cc} p & x_{i}=1\\ 1-p & x_{i}=0\\ 0 & otherwise \end{array},\right)$$

where 0 ≤ *p *≤ 1 and *t_j _*is dropped for simplicity of presentation. Under this definition, $N_{1}=\sum_{i=1}^{N}X_{i}$. Assuming that all cell states are independent, *N*_1 _has the binomial PMF given by

(2) $$P_{N_{1}}\left(n_{1}\right)=\left(\begin{array}{c} N\\ n_{1} \end{array}\right)p^{n_{1}}{\left(1-p\right)}^{N-n_{1}}$$

When the number of cells per well is large, say *N *> 100, the PMF of *N*_1 _at any given time instant can be accurately approximated by the Gaussian distribution due to the central limit theorem. Next we show that *N*_1_(*t*) is a Gaussian process.

**Proposition 1**. *The random process N*_1_(*t*) *is approximately Gaussian when the number of cells per well is large*.

*Proof*. At the beginning of the experiment, *t*_0_, *N*_1_(*t*_0_) is a Gaussian random variable. For any sampling point, at time *t_j_*, *N*_1_(*t*_j_) can be expressed as

(3) $$N_{1}\left(t_{j}\right)=N_{1}\left(t_{j-1}\right)+\Delta N_{1}\left(t_{j}\right)$$

where *N*_1_(*t_j_*_−1_) is the total number of shifted cells at time *t_j_*_−1_, and the additional number of shifted cells in the time interval [*t_j_*_−1_, *t_j_*] is given by

(4) $$\Delta N_{1}\left(t_{j}\right)=\overset{N-N_{1}\left(t_{j-1}\right)}{\underset{i=1}{\sum }}X_{i}$$

If *N *− *N*_1_(*t_j_*_−1_) is sufficiently large, *N *− *N*_1_(*t_j_*_−1_) > 32, then Δ*N*_1_(*t_j_*) is well approximated by a Gaussian random variable. Since *N*_1_(*t*_0_) is Gaussian, *N*_1_(*t_j_*) is Gaussian as well by mathematical induction. □

#### Modeling the cell shifting process

From our previous experimental observation, the cell shifting process on colon cancer cell-line HCT116 with a reporter for the MKI67 gene under lapatinib treatment shows a binary shifting characteristic. It is assumed that the number of shifting cells is related to: (i) the drug effect corresponding to different dosages; and (ii) the number of proliferating cells (non-shifted cells, *N *− *N*_1_). Since *N*_1_(*t*) is Gaussian process when the number of cells per well is large and *N *is a constant, the percentage of cells shifted at time *t *after applying drug, *ρ*_1_(*t*) = *N*_1_(*t*)/*N*, is a Gaussian process normalized to 0[1]. Similarly, for the control group, *ρ*_1*c*_(*t*) = *N*_1*c*_(*t*)/*N_c_*, is also a Gaussian process normalized to 0[1]. Then *ρ*(*t*) = *ρ*_1_(*t*) − *ρ*_1*c*_(*t*), the calibrated percentage of cells shifted at time *t *after applying drug, is a Gaussian process too. We are interested in the distribution of *ρ*(*t*), specifically, how the mean value of *ρ*(*t*), *ρ_av_*(*t*), changes along time under different dosage. Based on the above discussions, we propose the following model for cell shifting:

(5) $$\frac{d\rho_{av}}{dt}=\left(\gamma_{1}^{u}+\mu_{1}\right)\left(1-\rho_{av}\right)-\left(\beta +\mu_{2}\right)\rho_{av}+\nu$$

where $\gamma_{1}^{u}$ is the drug effective coefficient depending on the dosage *d*, and *β *> 0 is a balancing factor. *ρ_av_*(*t*) changes along time since the corresponding random process *ρ*(*t*) is non-stationary, thus its mean changes with time. Specifically, the change of *ρ_av_*(*t*) follows a linear differential equation (Eq.(5)) that reflects the fact that the change would be positively affected by the product of drug effectiveness and the percentage of cells not shifted (1st term in Eq.(5)), and negatively affected by the percentage of cells already shifted (2nd term in Eq.(5)), thus the term "balancing factor" for *β *since more shifted cells mean less non-shifted cells that the drug may affect.

In this model, we assume that both $\gamma_{1}^{u}$ and *β *change along time, thus the proposed model is a time-varying system. It is also assumed that the number of non-shifted cells, *N *− *N*_1_, decreases exponentially with the factor $\gamma_{1}^{u}$. $\mu ={\left[\mu_{1}\;\mu_{2}\right]}^{T}$ and *ν *are independent Gaussian white noise processes. *µ *represents the process noise. Its covariance matrix is

$$E\left[\mu \left(n\right)\mu^{T}\left(k\right)\right]=\left\{\begin{array}{cc} Q\left(n\right), & n=k\\ 0, & n\neq k \end{array}\right)$$

*ν *is the measurement noise. Its covariance matrix is

$$E\left[\nu \left(n\right)\nu \left(k\right)\right]=\left\{\begin{array}{cc} R\left(n\right), & n=k\\ 0, & n\neq k \end{array}\right)$$

The noise terms account for the various uncertainties introduced by the experiment. For instance, the cells may not be at the same cell cycle during the experiments, and thus may not be affected by the drug if some of the cells are actually dormant. This kind of uncertainties are modeled by process noise *µ*. There also exists another type of uncertainty due to measurement procedures, such as the imperfect photographic device and the image processing software. This type of uncertainty is modeled by measurement noise *ν*.

To observe the relationship between the drug effect coefficient $\gamma_{1}^{u}$ and the dosage *d*, we need to estimate $\gamma_{1}^{u}$ for each dosage. Since this is a time-varying model, $\gamma_{1}^{u}$ changes with time.

#### System identification from time-series data using Kalman filter

Kalman filtering [48] provides minimum-mean-square-error estimation of the state of a stochastic linear system disturbed by Gaussian white noise. In our proposed scheme, a Kalman filter is applied to estimate the coefficients, $\gamma_{1}^{u}$ and *β*, of the proposed cell shifting model. The corresponding state and measurement equations are

(6) w(n)=w(n-1)+μ(n-1)

(7) δ(n)=C(n)w(n)+ν(n)

where the 2-dimensional state vector (containing the parameters to be estimated) is $w={\left[\gamma_{1}^{u}\beta \right]}^{T}$. *δ *can be calculated as $\delta \left(n\right)=\frac{\rho_{av}\left(n+1\right)-\rho_{av}\left(n\right)}{\Delta t}$. $C=\left[1-\rho_{av}-\rho_{av}\right]$.

The implementation of the Kalman filter is given by the following equations [48]:

(8) $${ŵ}^{-}\left(n\right)={ŵ}^{+}\left(n-1\right)$$

(9) $$P^{-}\left(n\right)=P^{+}\left(n-1\right)+Q\left(n-1\right)$$

(10) $${ŵ}^{+}\left(n\right)={ŵ}^{-}\left(n\right)+K\left(n\right)\left[d\left(n\right)-C\left(n\right){ŵ}^{-}\left(n\right)\right]$$

(11) $$K\left(n\right)=P^{-}\left(n\right)C^{T}\left(n\right){\left[C\left(n\right)P^{-}\left(n\right)C^{T}\left(n\right)+R\left(n\right)\right]}^{-1}$$

(12) $$P^{+}\left(n\right)=P^{-}\left(n\right)-K\left(n\right)C\left(n\right)P^{-}\left(n\right)$$

where *K*(*n*) is the Kalman filter gain and *P *is the covariance matrix of the error. The superscripts ^- ^and ^+ ^indicate the *a priori *and *a posteriori *values of the variables, respectively. ${ŵ}^{-}$ and ${ŵ}^{+}$ are the prior and posterior estimates, respectively. *Q *and *R *are the covariance matrices of the parameter noise and external noise, respectively. The initial conditions are ŵ(0|δ0)=E[ŵ(0)] and $P_{0}=E\left[w\left(0\right)w^{T}\left(0\right)\right]$.

In general, a Kalman filter may be interpreted as a one-step predictor with an appropriate gain calculator [49]. Specifically, Eq.(10) is the one-step predictor, Eq.(11) calculates the Kalman filter gain, and Eq.(12) solves the corresponding Riccati equation.

Convergence of the Kalman filter is an important issue [48]. The rate of convergence is defined as the number of iterations to obtain the optimum estimates. The convergence of the Kalman filter includes the convergence of the estimates ŵ(n)and the convergence of the estimation error *e*(*n*). Convergence will be studied in detail in the simulations.

In practice, noise statistics (such as the covariance matrices) may not be known and need to be estimated. The Kalman filter is sensitive to the estimation error of noise statistics. Poor estimates of the noise covariance can result in filter divergence. An alternative would be using an *H_∞ _*filter [50,51].

## Results

Two-step analysis is performed to evaluate the drug effect study for different dosages. Firstly, we performed a proof-of-concept experiment using Monte Carlo simulation to demonstrate that the proposed model can mimic experimental observation. Secondly, we analyzed the time-varying drug effect for different dosages based on real experimental data from Dr. Bittner's lab at Translational Genomics Research Institution (TGen).

### Proof-of-concept experiment using Monte Carlo simulation

**Figure 4:**
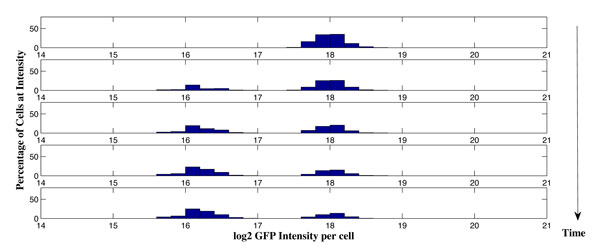
**The change of histogram of percentage of cells at intensity [2^14^, 2^19^] under drug intake along time using Monte Carlo simulation**.

It is assumed that a group of 200 cells has mean GFP intensity at 2^18^. When the drug is applied, each cell determines whether to shift to a lower intensity or not individually by flipping a coin (Bernoulli trial) at each time point, as we assumed in the theoretical model. The histograms of percentage of cells at intensity in the range of [2^14^, 2^19^] along time are shown in Figure 4. It is observed that the resulting histograms from the Monte Carlo simulation of the theoretical model match the measurement results from the TGen experiments performed on the cell-line. This demonstrates that the cell shifting is probably a binary decision, which lays the ground for our proposed theoretical model where a group of cells' decision can be modeled as binomial and can be closely approximated by Gaussian distribution when the number of cells is large.

### Drug effect analysis for the dosing study performed at TGen

**Figure 5:**
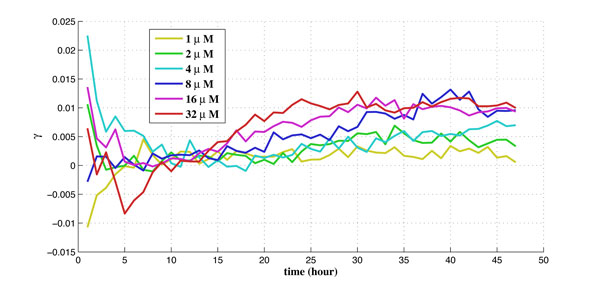
**The estimate of the drug effect coefficient along time for 6 different dosages**.

**Figure 6:**
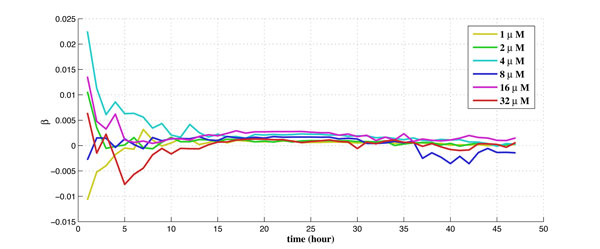
**The estimate of the balancing factor along time for 6 different dosages**.

For the experiments performed on the cell-line at TGen, there are 6 different dosages tested for the drug laptinib, from 1*µM *to 32*µM*. There are 6 biological duplicates for each dosage and each biological duplicate contains 200 to 400 cells. The obtained experimental data set contains time-series data of the intensity readings for each cell per hour along a 48-hour period. There are also corresponding experimental data set of the control group (without drug) for the purpose of calibration. The calibrated percentage of shifted cells is used as measurement data in the proposed algorithm using Kalman filtering. The obtained estimates of the drug effect coefficient $\text{(}\gamma_{1}^{u})$ and the balancing factor (*β*) along 48 hours for 6 different dosages are shown in Figure 5 and Figure 6, respectively.

It is observed from Figure 5 that in general the drug effect coefficient $\text{(}\gamma_{1}^{u})$ increases with the applied dosage, as expected. It seems that there exist certain thresholds for $\gamma_{1}^{u}$. For instance, $\gamma_{1}^{u}$ is much bigger with the dosages above 8*µM*. It is also observed that $\gamma_{1}^{u}$ increases with time as well. This reveals the time varying nature of the drug effect. Furthermore, Figure 5 shows that higher dosage corresponds to faster response time, e.g., $\gamma_{1}^{u}$ increases earlier and faster for higher dosage starting at ~10 hour. It is worth pointing out that, ideally, the percentage of shifted cells should be more than that in the control group without drug input, i.e., 0 ≤ *ρ*(*t*) ≤ 1. However, due to uncertainties and noise in the experiments, we actually observe that *ρ*(*t*) may be negative, especially during the first ~10 hours, before the drug is in effect.

Unlike $\gamma_{1}^{u}$, it is observed in Figure 6 that *β *remains roughly flat along time for a given dosage, because *β *is the balancing factor and should not change with time. However, *β *is different for different applied dosage, since higher dosage requires a higher balancing factor to maintain stability of the system. Again, the uncertainties and noise may dominate the system during the first ~10 hours (before the drug is in effect).

**Figure 7:**
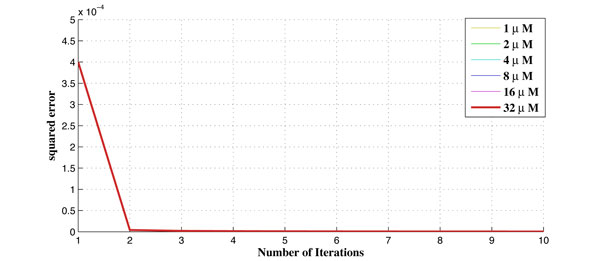
**The Convergence result of the the proposed algorithm using Kalman filter**.

Figure 7 shows the convergence of the Kalman filter. It converges in a few iterations in all cases.

### Post data processing for the dosing study performed at TGen

**Figure 8:**
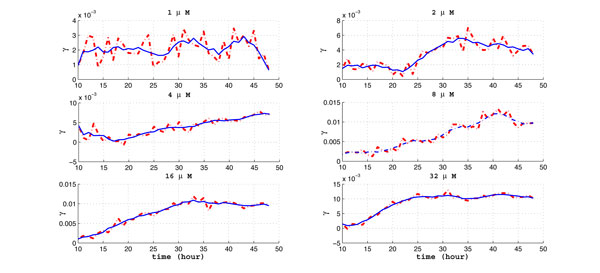
**The smoothed drug effect coefficient along time for 6 individual dosage**.

**Figure 9:**
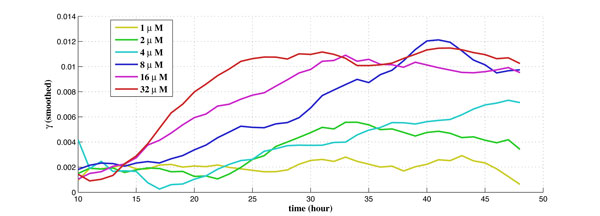
**The smoothed drug effect coefficient along time for 6 different dosages**.

**Figure 10:**
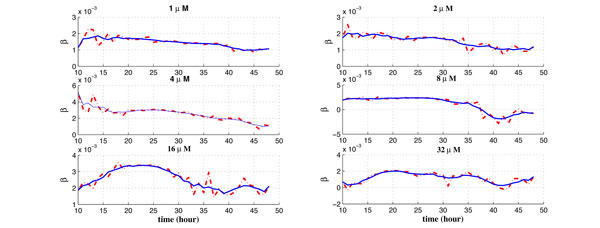
**The smoothed balancing factor coefficient along time for 6 individual dosage**.

**Figure 11:**
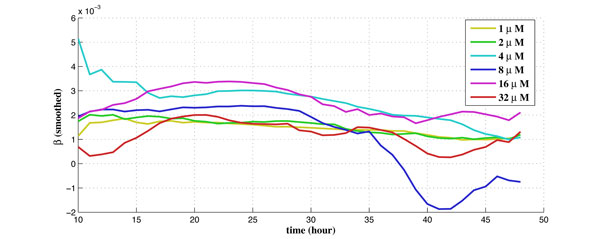
**The smoothed balancing factor coefficient along time for 6 different dosages**.

From Figure 5 and Figure 6, it is observed that drug effect $\text{(}\gamma_{1}^{u})$ and the balancing factor (*β*) is very "jittery," especially for the initial ~10 hours. Such a phenomenon may result from experimental noise, or that the cells may need certain "commitment time" after the drug is added. In order to better compare the drug effect for different dosages, we smooth the results and only take into account data after the first 10 hours. We apply a moving-average filter with filter coefficients determined by an unweighted linear least-squares regression and a 2nd-degree polynomial model. The span for the moving average is 5. Figure 8 shows the smoothed drug effect coefficient $\text{(}\gamma_{1}^{u})$ along time for 6 individual dosages. It can be observed that the drug effect is more jittery for small dosages, such as 1*µ*M. The smoothed $\gamma_{1}^{u}$ along time for 6 dosages are compared in Figure 9. It is observed that there exists a "plateau" $\text{(}\gamma_{1}^{u}\approx 0.01)$ for higher dosages above 8*µ*M. The plateau is reached at 38 hours, 30 hours, and 24 hours, for dosages 8*µ*M, 16*µ*M, and 32*µ*M, respectively. The smoothed balancing factor (*β*) for individual dosage can be found in Figure 10, and the smoothed *β *for 6 dosages are compared in Figure 11.

## Conclusions and future work

The ultimate goal of target-based cancer drug development is to improve the efficacy and selectivity of cancer treatment by exploiting the differences between cancer cells and normal cells. The current cancer drug development process is confronting huge challenges, such as how to better understand the target in context and develop predictive preclinical models to better understand the molecular mechanisms of the biological systems they target and hence reduce the attrition rate. An integrated experimental and theoretical approach is proposed to assess the efficacy of molecularly targeted agents based on cell-line platforms. As a first step, drug efficacies for different dosages are characterized along time. Specifically, tumor cell's responses are analyzed through the use of fluorescent reporters sampled frequently over a time course; quantification is done by microscopic scanning of cells in culture in multi-well plates using the automated epifluorescent imager; fluorescent intensity data are extracted from these images using specialized large-scale image analysis tools developed for this application; the dynamics of drug efficacy for different dosages are studied using dynamic modeling; and time-varying parameters are estimated using system identification techniques. It is observed that the drug efficacy is time and dosage dependent. The objectives are two-fold: (i) The dosing study for MTAs should be based on both efficacy and toxicity consideration to find the biologically effective dose (BED) instead of the maximum tolerated dose (MTD) for cytotoxic agents. The time course of drug effect for different dosages can provide information on the gradient of drug effect vs. dosage, and thus on the BED. (ii) Instead of a fixed time point pharmacodynamics study of MTA, characterization of the entire time course of drug effect provides insight into designing an optimal schedule for drug administration.

Based on a similar experimental set-up and measurements to follow the cell/drug (dosages) dynamics, a truly multi-dimensional dynamics of tumor cell responses to drugs can be characterized through systematic perturbations to test different combinations of cell types, reporters, and drugs/dosages, augmented by iterative systematic theoretical analysis. Such an approach would facilitate the study of optimal dose and schedule, such as whether short pulses of higher dose, persistent dosing with lower dose, or some other regimen would have the most favorable outcomes. Moreover, the complex target context can be inferred with multi-dimensional cell response dynamics with the help of advanced system identification methods. In sum, better intervention strategies can be designed. Such topics are either currently being pursued or will be in future projects.

## Competing interests

The authors declare that they have no competing interests.

## Authors' contributions

XL and LQ developed and implemented the algorithm, conducted all simulations and data processing and wrote the initial draft of the paper. JH performed the image analysis. MB performed the experiments. ED advised XL on algorithm development and revised the paper. All authors read and approved the final manuscript.
